# Is hypnotic assessment relevant to neurology?

**DOI:** 10.1007/s10072-022-06122-8

**Published:** 2022-05-13

**Authors:** Lorenzo Fontanelli, Vincenzo Spina, Carmelo Chisari, Gabriele Siciliano, Enrica L. Santarcangelo

**Affiliations:** 1 Department of Clinical and Experimental Medicine, University of Pisa, Pisa, Italy; 2 Department of Translational Research NTMS, University of Pisa, Pisa, Italy

**Keywords:** Hypnosis, Rehabilitation treatments, Hypnotizability scale, Motor imagery

## Abstract

Studies conducted in healthy subjects have clearly shown that different hypnotic susceptibility, which is measured by scales, is associated with different functional equivalence between imagery and perception/action (FE), cortical excitability, and information processing. Of note, physiological differences among individuals with high (highs), medium (mediums), and low hypnotizability scores (lows) have been observed in the ordinary state of consciousness, thus independently from the induction of the hypnotic state, and in the absence of specific suggestions. The potential role of hypnotic assessment and its relevance to neurological diseases have not been fully explored. While current knowledge and therapies allow a better survival rate, there is a constant need to optimize rehabilitation treatments and quality of life. The aim of this paper is to provide an overview of hypnotizability-related features and, specifically, to discuss the hypothesis that the stronger FE, the different mode of information processing, and the greater proneness to control pain and the activity of the immune system observed in individuals with medium-to-high hypnotizability scores have potential applications to neurology. Current evidence of the outcome of treatments based on hypnotic induction and suggestions administration is not consistent, mainly owing to the small sample size in clinical trials and inadequate control groups. We propose that hypnotic assessment may be feasible in clinical routine and give additional cues into the treatment and rehabilitation of neurological diseases.

## Introduction

Hypnotizability is a psychophysiological trait predicting the proneness to modify perception, memory, and behavior according to specific suggestions, and to enter the hypnotic state. It is measured by scales [1, 2], is substantially stable through life [3], and is characterized by cerebral and cerebellar morpho-functional peculiarities [4, 5]. The various hypnotizability scores are also associated with different cortical dopaminergic, serotoninergic, and gabaergic tone and oxytocin availability [6]. In the cognitive-emotional domain, high hypnotizability is associated with greater attentional stability/deeper absorption [7], greater fantasy and expectancy proneness, acquiescence and consistency motivation, and emotional intensity [8]. Among the physiological correlates, which are observable also out of hypnosis (that is in the ordinary state of consciousness) and in the absence of specific suggestions, with respect to the persons scoring low on hypnotizability scales (lows), highly hypnotizable individuals (highs) display less close postural and visuomotor control, pre-eminent parasympathetic control of heart rate, less impaired post-occlusion flow-mediated dilation (FMD) of peripheral arteries during mental stress and nociceptive stimulation [2], paradoxical pain control by transcranial anodal stimulation of the cerebellum [9], lower interoceptive accuracy, and greater interoceptive sensitivity 10.

In this scoping review, we propose that hypnotic assessment can assist in the prognosis and treatment of a few neurological diseases. With respect to lows and medium hypnotizables (mediums), in fact, highs display stronger functional equivalence between imagery and perception/action (FE) and different modes of cortical information processing [11, 12], greater excitability of the motor cortex during resting and sensori-motor imagery conditions [13, 14], greater proneness to modulate the activity of the immune system [15–20] and peculiar abilities to control pain through imagery, despite the low efficacy of their opioid system [21, 22]. Although highs represent 15% of the entire population [23], mediums often exhibit physiological characteristics intermediate between highs and lows [13, 21], thus being able to take partial advantage from suggestions and hypnotic treatments. Studies aimed at improving their imagery abilities are in progress [24].

## Functional equivalence between imagery and perception/action, information processing

The term “functional equivalence” (FE) refers to the similar brain activation occurring during actual and imagined perception/action [25, 26]. As previously mentioned, FE is stronger in highs than in lows [10, 11] and this does not depend on expectation and/or voluntary control. Behavioral investigations, in fact, have revealed that highs display the same earlier component of the vestibulo-spinal reflex when the head is physically rotated toward one side and when this posture is imagined. Since the earlier component of the vestibulospinal reflex is not controlled by expectation and volition, it was hypothesized that brain circuits were similarly activated in the two conditions. This hypothesis was supported by topological analyses of the EEG signals which showed that the global asset of brain activities during actual and imaginatively rotated posture of the head were significantly more similar between each other in highs than in lows [12]. Theoretically, together with the highs’ greater motor cortex excitability [13], these hypnotizability-related characteristics could make highs more prone than the general population to take advantage from imagery training and brain-computer interface (BCI) interventions [27].

Highs and lows differ also in the mode of information processing. Spectral analysis of EEG signals recorded during motor imagery failed to detect significant differences between basal and imagery conditions in highs, in contrast to lows ^28^. Topological analyses revealed that during sensori-motor imagery lows display significant task-related changes in specific areas, whereas highs exhibit largely distributed, very small changes in their brain activity with respect to resting conditions [12, 28]. In other words, in highs, the cortical representation of both motor action and motor imagery involves smaller changes across larger regions. On this basis, we hypothesize that the highs’ mode of information processing could make them more susceptible than lows’ to recovery after neurological injuries. It has been also shown that hypnotic sessions influence brain plasticity [29], thus theoretically increasing the odd of recovery hypothesized for highs’ owing to their greater FE[12] and to the greater excitability of their motor cortex [13].

Overall, experimental findings obtained in healthy participants suggest that being highly hypnotizable may be a favorable prognostic factor for imagery training aimed at neurorehabilitation. Current literature supports this hypothesis and indicates that imagery training with and without hypnosis is a useful complementary intervention in several movement disorders not only of functional origin [30]. Several studies and meta-analyses, in fact, show that in chronic post-stroke patients mental imagery is more efficacious than standard physical therapy [31], improving gait, balance [32], and motor learning [33]. Moreover, the ability to visualize upper limb mental motor images correlates with post-stroke movement, functionality, and strength, and mental visualization of motor ability correlates with upper limb motor function [34]. Contrasting reports can be due to great heterogeneity between trials in intensity, duration, and studied variables.

In patients with multiple sclerosis (MS) guided imagery increases walking speed and distance, reduces fatigue, improves the quality of life, and positively influences dynamic balance and perceived walking ability [35, 36]. Imagery has also been used to identify and manage emotional aspects of MS possibly associated with symptoms worsening [37] as well as to counteract fatigue and facilitate return-to-work [38]. Fatigue is one of the greatest burdens for people with MS, is present in most of them, and is considered one of the worst symptoms by about half of patients, often disabling and interfering with daily life activity. Different drugs have been proposed to reduce fatigue in patients with MS but have shown poor efficacy. Since MS fatigue has a central origin, with evidence of abnormal cortical activation during voluntary movements [39], imagery training could improve the treatment outcome. Nonetheless, although people with MS have preserved MI ability, impairments in the speed and accuracy of imagery tasks (but, interestingly, not in the vividness of MI) have been found, compared to healthy controls [40]. In this respect, being highs may be greatly helpful [12, 13]. Recently a blinded, randomized trial directed to patients with MS assessed the effects of a telerehabilitation-based motor imagery training (Tele-MIT). Tele-MIT significantly enhanced walking speed, dynamic balance during walking, balance confidence and perceived walking ability, improved quality of life, and reduced fatigue, anxiety, and depression [41]. Studies conducted in small samples and/or patients with low compliance not reporting beneficial effects of MI cannot be considered contrasting with the idea that MI can improve the daily life of patients with MS [42].

In Parkinsonian (PD) patients, motor imagery of gait in gait freezers improved gait velocity, stride length, stance time, swing time, single support time, and total double support time [43]. Dynamic Neuro-Cognitive Imagery (DNI™)—a codified method for imagery training—improved balance, walking, mood, and coordination, and patients felt more physically and mentally active [44]. Autogenic training—which is, like auto-hypnosis, aimed at achieving relaxation—improved PD symptoms [45] as well as cervical dystonia [46].

Also in amyotrophic lateral sclerosis (ALS) a 4-week hypnosis-based treatment consisting of a hypnosis induction followed by a core phase of guided visual imagery with patient-tailored suggestions and a conclusive phase aiming at self-hypnosis, not only improved wellbeing and quality of life and reduced depression (also in the caregivers), but was even associated with a slower functionality loss at a 6-month follow-up, in a study with fifteen patients compared to fifteen one-to-one matched controls [47].

## Immune system

A meta-analysis including 51 studies showed that hypnosis is effective in the control of autoimmune diseases [48]. Derangement of the immune system is found in a few neurological diseases. In MS the immune activity damages the myelin sheath around the axons, causing axonal damage and disruption in the propagation of nerve impulse. In the most frequent form of MS, named “relapsing–remitting MS,” the immune system is intermittently activated by disparate triggers causing exacerbations of the disease. Among various triggers (e.g., infections, post-partum…), psychological stress has been considered both for the onset and for relapse [49]. In this respect, relaxation training, which is more effective in highs [2], can counteract stress effects, and imagery has been used also to manage emotional aspects of MS possibly associated with symptoms worsening [37].

Another chronic neurological autoimmune disorder is myasthenia gravis (MG), in which, in the usual forms, autoantibodies are directed against acetylcholine receptors (AChR) or muscle-specific kinase receptors (MuSK) impairing the proper formation of end-plate potential in the muscle fibers and, thus, muscle contraction. In a recent study, patients affected with MG and a higher level of depression or stress had also a higher level of relapse rate, suggesting that emotional factors may influence the course of MG [50]. Thus, also MG treatment could be integrated by relaxation and imagery training and, theoretically, the outcome could be predicted by hypnotic assessment. The same may be suggested for other, rarer, autoimmune diseases such as nervous system vasculitis, optic neuritis, and neurosarcoidosis, a condition where inflammatory infiltrates are found throughout the nervous system.

Hypnotic treatments may influence the immune system by modulation of the autonomic activity. The highs’ higher parasympathetic tone during relaxation, with respect to lows ^2^ and their ability to further increase it after hypnotic induction [51] could enable them to improve the activity of the immune system through counteracting the sympathetic inhibition on humoral and cell immunity. Highs are expected to be more prone than lows and mediums to take advantage from positive/pleasant/relaxing imagery [2], inducing changes in the immune activity owing to their peculiar imagery abilities, i.e., stronger FE and deeper absorption in mental activities [7]. After imagery and relaxation training, indeed, highs show greater decreases than lows in the activity of Natural Killers lymphocytes and lymphocyte proliferative response [15, 16, 20]. Gruzelier et al. [52] showed that 10 sessions of hypnosis buffer the decline in NKP, CD8, and CD8/CD4 ratio occurring in students during examination sessions and increase cortisol levels. Also, effects on B-cells and helper T cells have been reported [16, 17] together with upregulation of the expression of immune-related genes in lymphocytes by hypnosis [18]. More recently, autogenic training increased the IgA levels in patients undergoing surgery for breast cancer [19]. In geriatric patients, autosuggestions improved quality of life, increased serum cortisol and interleukin-6 in geriatric patients who displayed also tendency to increase interleukin-2, interferon-γ, and N-acetylaspartate/creatine ratio in the prefrontal cortex [53].

In brief, a multidisciplinary approach including suggestions for relaxation, pleasant experiences, and/or motor imagery may improve the treatment outcome, which could be predicted by hypnotic assessment.

## Pain control: suggestions for analgesia or motor imagery, drugs selection

Suggestions for analgesia and/or pleasant imagery reduce pain whichever the pain origin and duration [54–56]. They can be personalized (for instance, “experiencing wellness and fitness on a green grass carpet while trees swing and the wind caresses the face,” or “lying on warm sand while listening to the see,” or “relaxing on a soft snow carpet looking at mountains”) and can refer or not to pain. In the latter case, for instance, the patients can be invited to imagine that “her/his pain leaves the abdomen and flies on clouds which become darker and darker,” or “to imagine that a particular glove prevents any unpleasant information related to the painful body part to reach the brain, as if nerves has been cut”). Of note, suggestions are effective also out of the hypnotic state, thus being available to clinicians in every situation [21, 22]. Moreover, there is a large amount of experimental evidence indicating that suggestions reduce pain not only in highs, but in the general population [21, 22], although through specific hypnotisability-related mechanisms in highs, suggestions-induced placebo in lows, and possibly mixed mechanisms in mediums. An important difference, in this respect, is that the highs’ response to the suggestions for analgesia does not involve the release of endogenous opioids, whereas placebo-related analgesia depends on opioids [21, 22].

Suggestions for analgesia and self-hypnosis have been efficaciously used to reduce pain in post-stroke patients, multiple sclerosis, amyotrophic lateral sclerosis Parkinson’s disease, Guillain-Barré syndrome, HIV, post-polio syndrome, diabetes, and cancer [57]. Moreover, hypnotic treatment improved the efficacy of self-hypnosis in MS patients [58], led to a reduction of about 90% in the median number of migraine attacks, and outperformed standard prophylactic treatment with prochlorperazine [59], even in juvenile migraine, where self-hypnosis was superior to propranolol and placebo in reducing headache frequency [60]. In patients with chronic tension headache, hypnotherapy reduced the number of headache days, the number of headache hours, headache intensity, and anxiety scores [61]. In both migraine and tension headache patients’, hypnosis and placebo induced similar improvements and patients reduced their medication usage. Of note, hypnosis has been effective in reducing migraine symptoms even in people with low susceptibility to hypnosis [62], which is likely due to placebo-related mechanisms induced by suggestions in mediums and lows [21, 22]. These studies did not show any difference between one and four hypnotic sessions indicating that even a short treatment may be effective.

In the general population, both physical stimulation and motor imagery (MI) are used to control pain, in line with the concept of functional equivalence between imagery and perceptions (see the “Functional equivalence between imagery and perception/action, information processing” section). MI, in fact, produces functional cerebral reorganization similar to those obtained by physical practice [63] and may reverse, at least partially, the alterations in the body schema observed in neuropathic pain and pain perception. In highs, the effects of motor imagery are expected to be stronger than in the general population owing to their stronger functional equivalence between imagery and perception/action [12] and to their greater proneness to neural plasticity after hypnotic treatments [29]. For instance, pain intensity associated with spinal injuries (SCI) and associated with a reorganization of the SI could be efficaciously treated by suggestions of analgesia as well as by motor imagery training and the outcome should be better in highs[64]. The same can be hypothesized for phantom limb pain (PLP)[65] and complex regional pain syndrome type 1 (CRPS1) [66], associated with a maladaptive neuronal plasticity not limited to SI, but extended to the dorsal ganglion [67].

A specific motor imagery protocol—graded motor imagery (GMI) consisting of three sequential phases (identifying whether pictures of part of the body depict the left or right side, imagining own movements, and performing movements of the unaffected side of the body)—has been used in several patients. A non-randomized controlled trial on twenty-eight patients with first-ever stroke [68] treated with twenty sessions of one hour each of GMI or conventional rehabilitation, showed that patients on GMI had better scores over the control group in the pain section of Fugl-Meyer Assessment (FMA), a stroke-specific index designed to assess motor functioning and sensation in patients with post-stroke hemiplegia. In patients with CRPS1 and PLP, a short GMI training reduced pain up to six months after the end of the program, improved function, and, limitedly to CRSP1 patients, reduced swelling compared to standard treatment [69–71]. Interestingly, intensive 6-week training in MI, either once a week or once a fortnight, consisting of “body scan” exercise and imagination of movements and sensations in the phantom limb, reduced cortical reorganization and pain in PLP patients. Of note, patients who undertook the three stages in different orders did not respond, suggesting that the effect of GMI is dependent from a specific, sequential activation of a variety of cortical networks[70]. In contrast, another multicentred study aimed at using GMI in clinical practice which included both CRPS type 1 and 2 (patients with evidence of nerve injury) failed to show any significant improvement in pain [72].

In tetraplegic patients, a modified GMI protocol [73] induced a significant pain reduction, on the numeric rating scale of pain and on visual analog scale of pain, for the mean total score at the Neuropathic Pain Symptom Inventory and for its subsections assessing dysesthesia, paraesthesia, allodynia, and pressor pain. Moreover, after MI intervention, patients reported better sleep and less interference of pain on sleep compared to the control group. In brief, suggestions for analgesia and pleasant imagery as well as motor imagery are useful tools for pain control. Nonetheless, Gustin et al. found that, in a cohort of SCI patients, imaging movements of ankle plantar and dorsiflexion increased pain in patients who suffered from below-level neuropathic pain and might even induce unpleasant sensations in those who were otherwise symptom free [74]. The authors suggest that this unexpected finding may be due to increased sodium channel expression or inhibitory dysfunction at the lesion level.

In brief, suggestions for analgesia and pleasant imagery as well as motor imagery are useful tools for pain control.

Finally, we must underline that the pharmacological treatments of acute/chronic pain can be oriented by hypnotic assessment. Highs, in fact, exhibit the µ1 polymorphism (A118G, rs1799971) associated with low sensitivity to opiates [21, 22]. Such polymorphism requires higher opiate dosages and makes highs more sensitive to side opiate effects than prone to the experience of analgesia. Thus, hypnotic assessment in pain patients is useful to orient pharmacological therapies.

## Limitations and conclusions

Imagery abilities such as the strength of the functional equivalence between imagery and perception/action, the proneness to control the immune system through relaxation by enhancing the parasympathetic tone, to reduce pain through cognitive strategies, and to take advantage from certain pharmacological pain, can be predicted by hypnotic assessment. Suggestions with and without induction of hypnosis also allow an improvement of the patients’ quality of life including physical symptoms and disease progression. Thus, we propose hypnotic assessment as a routine evaluation of neurological patients.

A limitation of the role of hypnotic assessment could be the fact that highs and lows represent 15% of the population each, while mediums represent its largest part [23]. Nonetheless, mediums often exhibit physiological characteristics intermediate between highs and lows [13, 21], thus being able to take partial advantage from suggestions and hypnotic treatments. Clinical studies should be conducted according to the recent guidelines for hypnosis research, which suggest to include mediums and explore both wake and hypnotic conditions with and without suggestions [75].

In conclusion, our opinion is that novel approaches to neurological patients suffering from sensory, motor, and cognitive symptoms of various origins should consider also hypnotizability among the classical psychophysiological traits potentially influencing symptoms and recovery and usually investigated in the context of the body-mind interaction.
